# Supplementary material for: Role of the osaA Transcription Factor Gene in Development, Secondary Metabolism and Virulence in the Mycotoxigenic Fungus Aspergillus flavus
Source: Toxins (Basel). 2025 Dec 30;18(1):23. doi: 10.3390/toxins18010023 (PMC12845751; doi:10.3390/toxins18010023)
Supplement: Supplementary file 1 [file toxins-18-00023-s001.zip › Supplementary Materials.pdf]

Supplementary Materials: Role of the *osaA* Transcription Factor Gene in Development, Secondary Metabolism and Virulence in the Mycotoxigenic Fungus *Aspergillus flavus*

Farzana Ehetasum Hossain, Apoorva Dabholkar, Jessica M. Lohmar, Matthew D. Lebar, Brian M. Mack and Ana M. Calvo

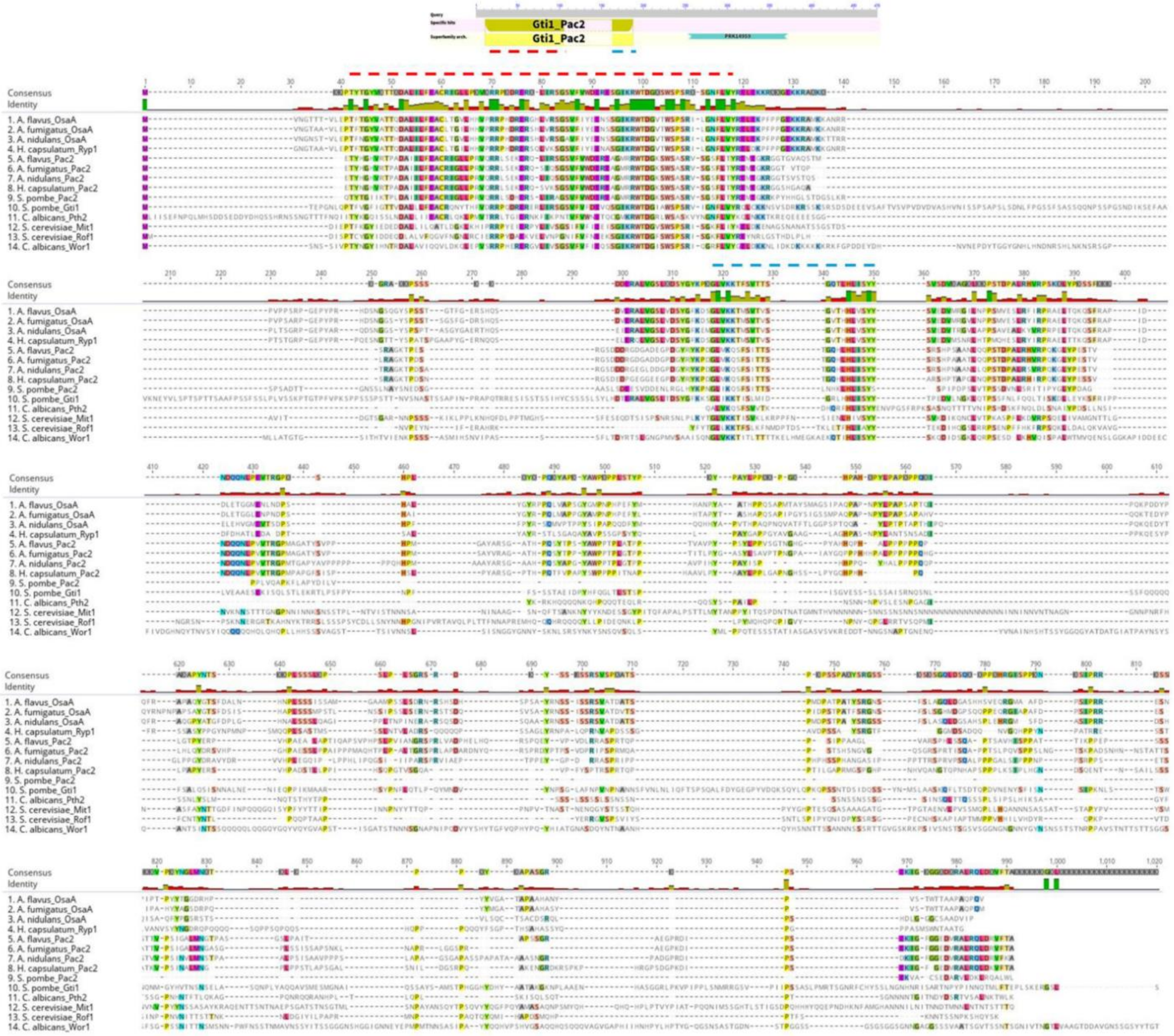

Figure S1: Multiseqeunce alignment of Gti1 and Pac2 orthologous proteins. Using Geneious software, a MAFFT multisequence alignment of Gti1 and Pac2 proteins from *A. fumigatus*, *A. flavus*, *A. nidulans*, *H. capsulatum*, *C. ablicans*, *S. cerevisiae*, and *S. pombe* was carried out using default settings to align the protein sequences based on their predicted Gti1/Pac2 (WOPR) protein domains. Two conserved regions within the WOPR domain were identified and designated as WOPRa (dashed red line) and WOPRb (dashed blue line).

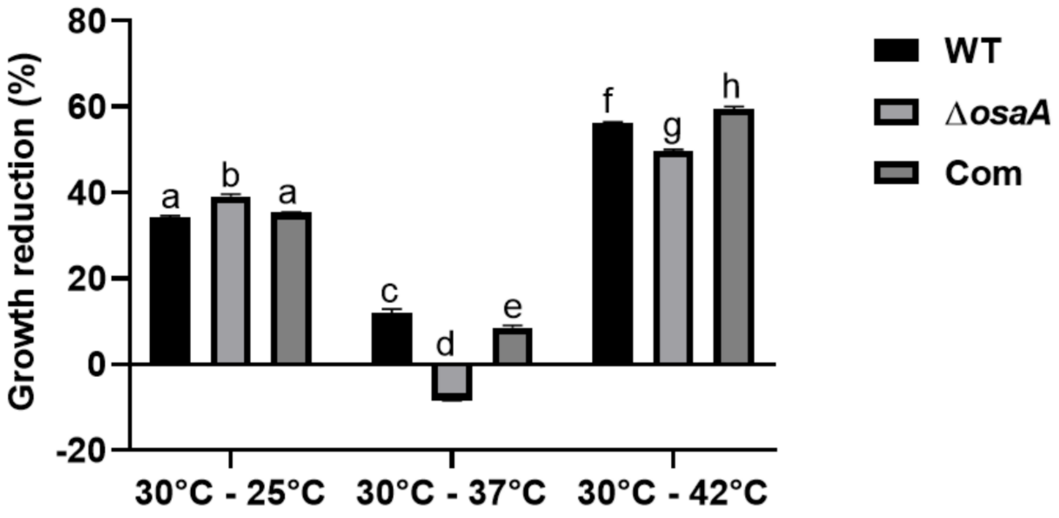

Figure S2. Role of *osaA* in temperature sensitivity in *A. flavus*. Wild type (WT), deletion *osaA* (Δ*osaA)* and complementation *osaA* (Com) were point-inoculated on PDA Medium. Cultures were incubated at 25°C, 30°C, 37°C and 42°C in the dark for 5 days . Colony diameter was measured after incubation. The experiment was carried out in triplicate. Data were represented as the percentage of reduction in the growth of strains on 30°C versus 25°C; 30°C versus 37°C and 30°C versus 42°C. Error bars represent the standard error. Columns with different letters represent values that are statistically different ($p < 0.05$), as determined by two-way ANOVA with Tukey test comparison.

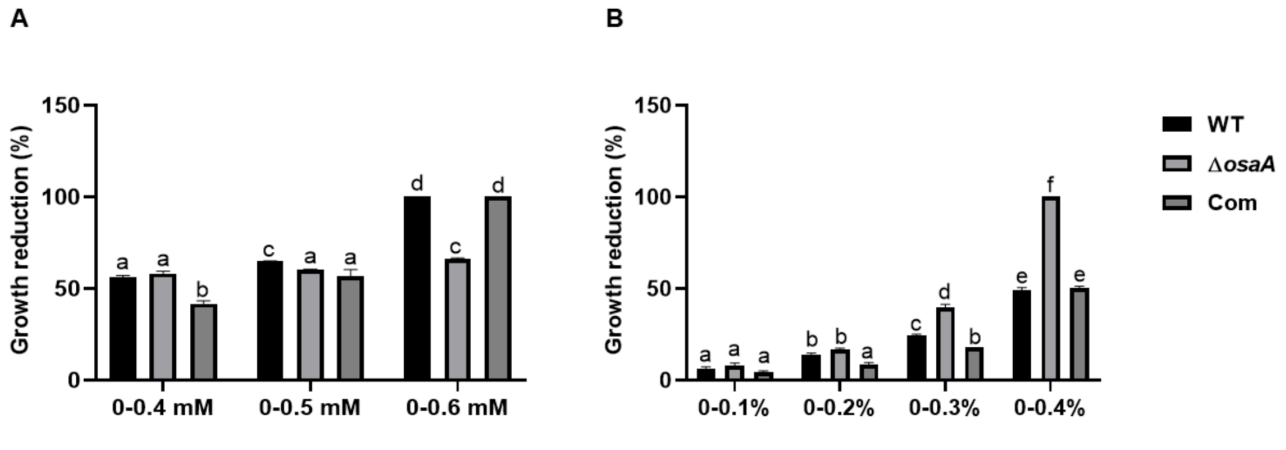

Figure S3. Role of *osaA* in oxidative stress sensitivity in *A. flavus*. **(A)** Wild type (WT), deletion *osaA* (Δ*osaA)* and complementation *osaA* (Com) were point-inoculated on PDA medium supplemented with 0, 4 ,5 and 6 mM menadione and incubated at 30°C for 3 days. Colony diameter was measured after incubation. The experiment was performed with three replicates. Data was represented as the percentage of reduction in the growth of strains on PDA (0 mM

menadione) versus cultures supplemented with 0, 0.4, 0.5 or 0.6 mM menadione. Error bars represent the standard error. Columns with different letters represent values that are statistically different ($p < 0.05$), as determined by two-way ANOVA with Tukey test comparison. **(B)** Wild type (WT), deletion *osaA* (Δ*osaA)* and complementation *osaA* (Com) were point-inoculated on PDA medium supplemented with 0, 0.1, 0.2, 0.3 and 0.4% hydrogen peroxide and incubated at 30°C for 3 days. Colony diameter was measured after incubation. The experiment was performed with four replicates. Data was represented as the percentage of reduction in the growth of strains on PDA (0% H_2O_2) versus cultures supplemented with 0.1, 0.2, 0.3 or 0.4% H_2O_2. Error bars represent the standard error. Columns with different letters represent values that are statistically different ($p < 0.05$), as determined by two-way ANOVA with Tukey test comparison.

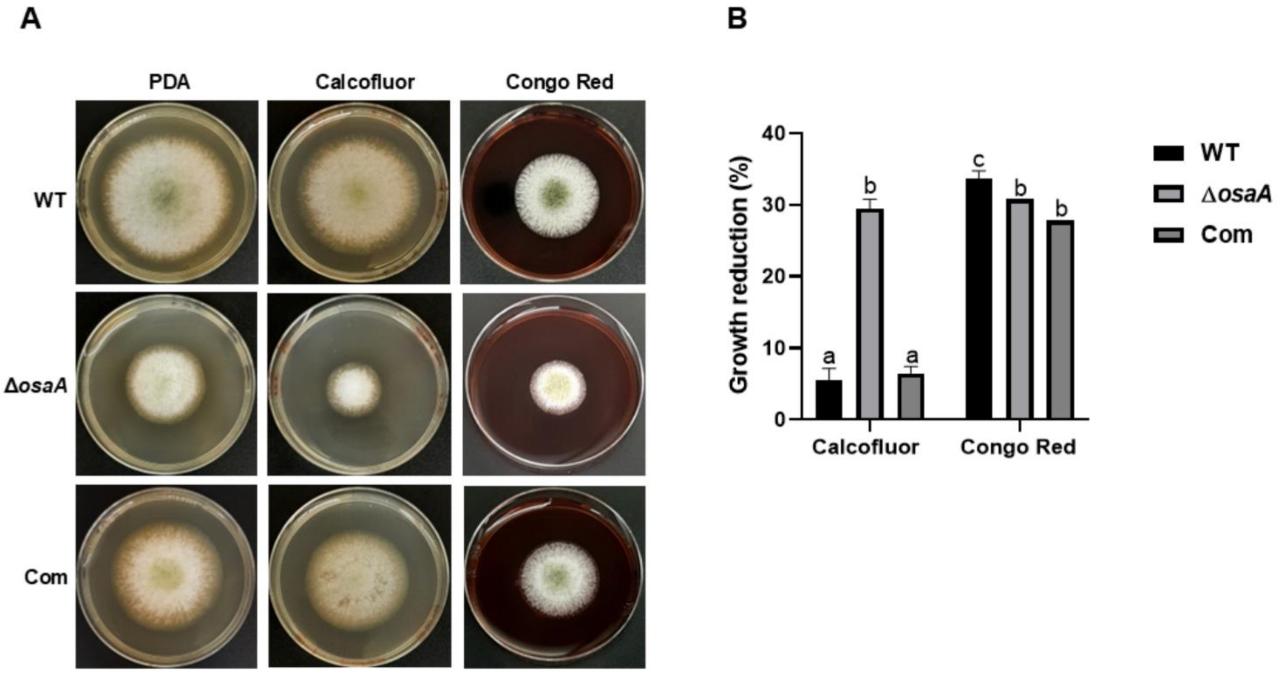

Figure S4. Role of *osaA* in cell wall integrity in *A. flavus*. **(A)** Wild type (WT), deletion *osaA* (Δ*osaA)* and complementation *osaA* (Com) were point-inoculated on PDA medium supplemented with Calcofluor white (0.1 mg/mL) and Congo Red (0.3 mg/mL) for cell wall stress and incubated at 30°C for 3 days under dark condition. Colony diameter was measured after incubation. The experiment was performed with three replicates. **(B)** Data were represented as the percentage of reduction in the growth of strains on PDA (without any supplement) versus cultures supplemented with Calcofluor White (0.1 mg/mL) or Congo Red (0.3 mg/mL). Error bars represent the standard error. Columns with different letters represent values that are statistically different ($p < 0.05$), as determined by two-way ANOVA with Tukey test comparison.

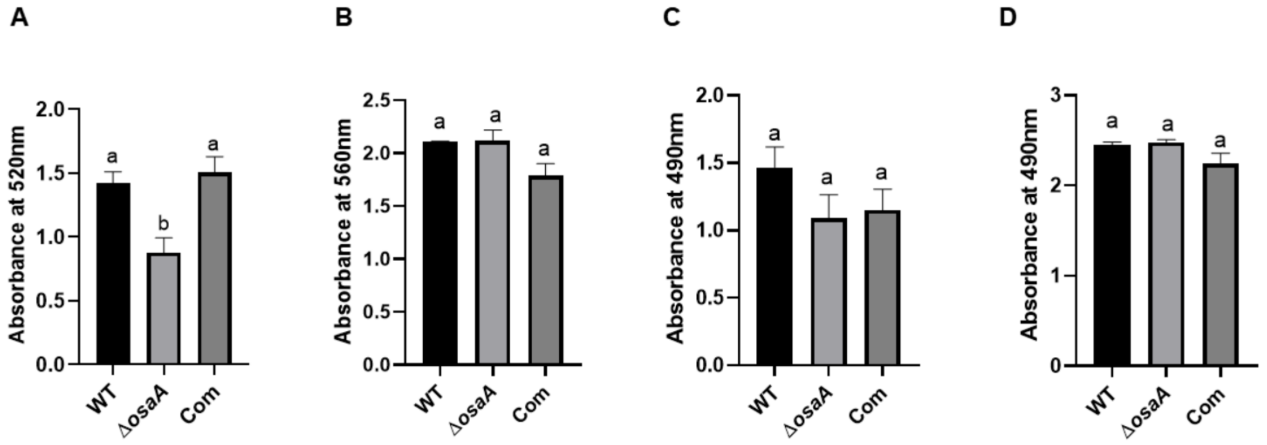

Figure S5. Role of *osaA* in the synthesis of cell wall components in *A. flavus*. Wild type (WT), deletion *osaA* (Δ*osaA)* and complementation *osaA* (Com) were grown at 30°C for 2 days at shaking culture at 250 rpm. Biomass were analyzed to measure the cell wall components **(A)** chitin, **(B)** mannoprotein, **(C)** insoluble glucan and **(D)** soluble glucan. The error bars represent standard error. Different letters on the columns indicate values that are statistically different ($P < 0.05$), as determined by one-way ANOVA with Tukey test comparison.

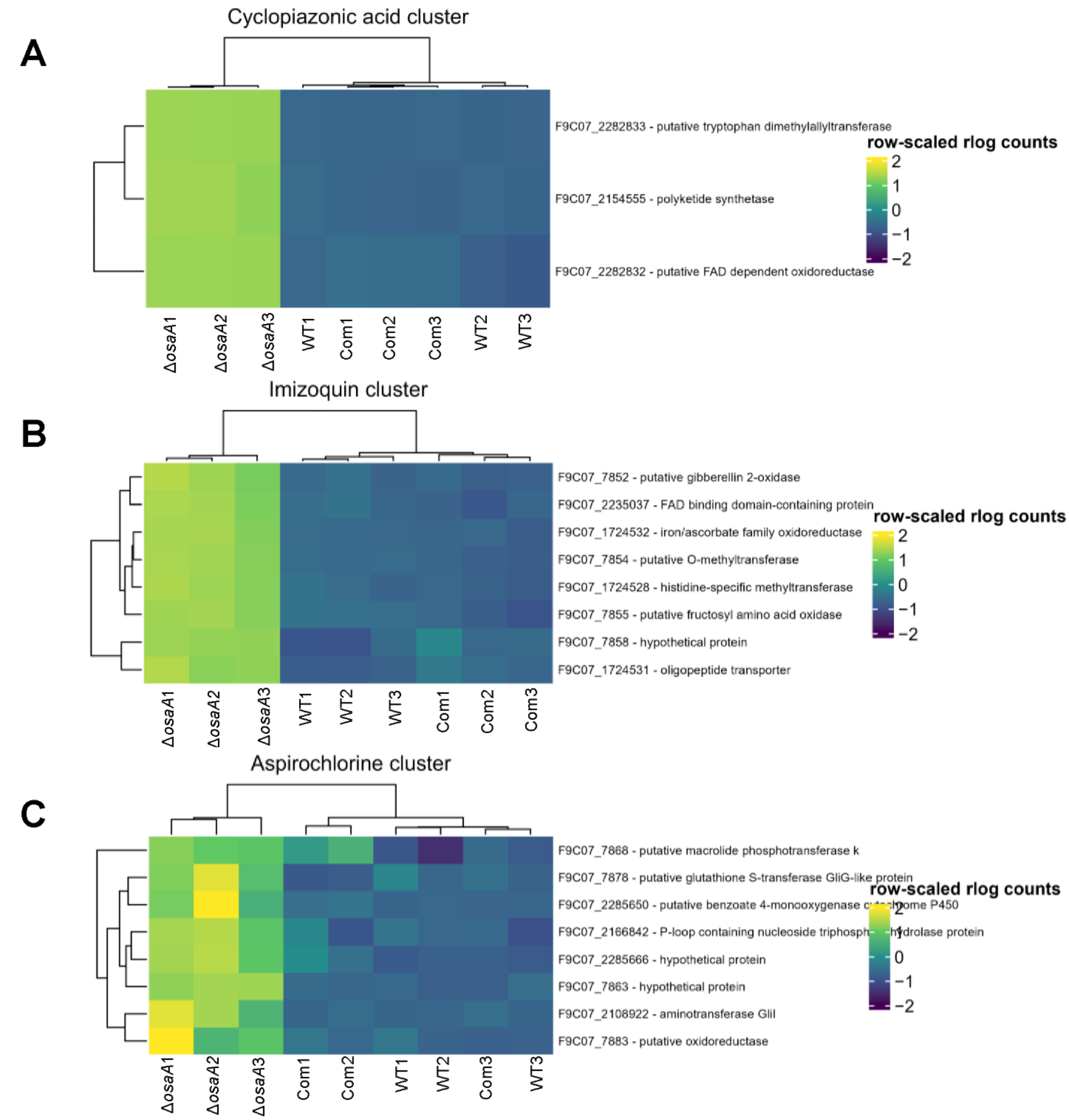

Figure S6. Differential expression of genes from the cyclopiazonic acid gene clusters, imizoquin genes and aspirochlorine gene**.** Heatmap representation of cyclopiazonic acid **(A)** imizoquin **(B)** and aspirochlorine **(C)** gene expression profiles in wild type (WT), *osaA* deletion and complementation strains. Each row corresponds to a gene, annotated by its locus tag and predicted function. Yellow indicates higher expression while dark blue indicates lower expression, with color intensity reflecting the magnitude of fold change values.

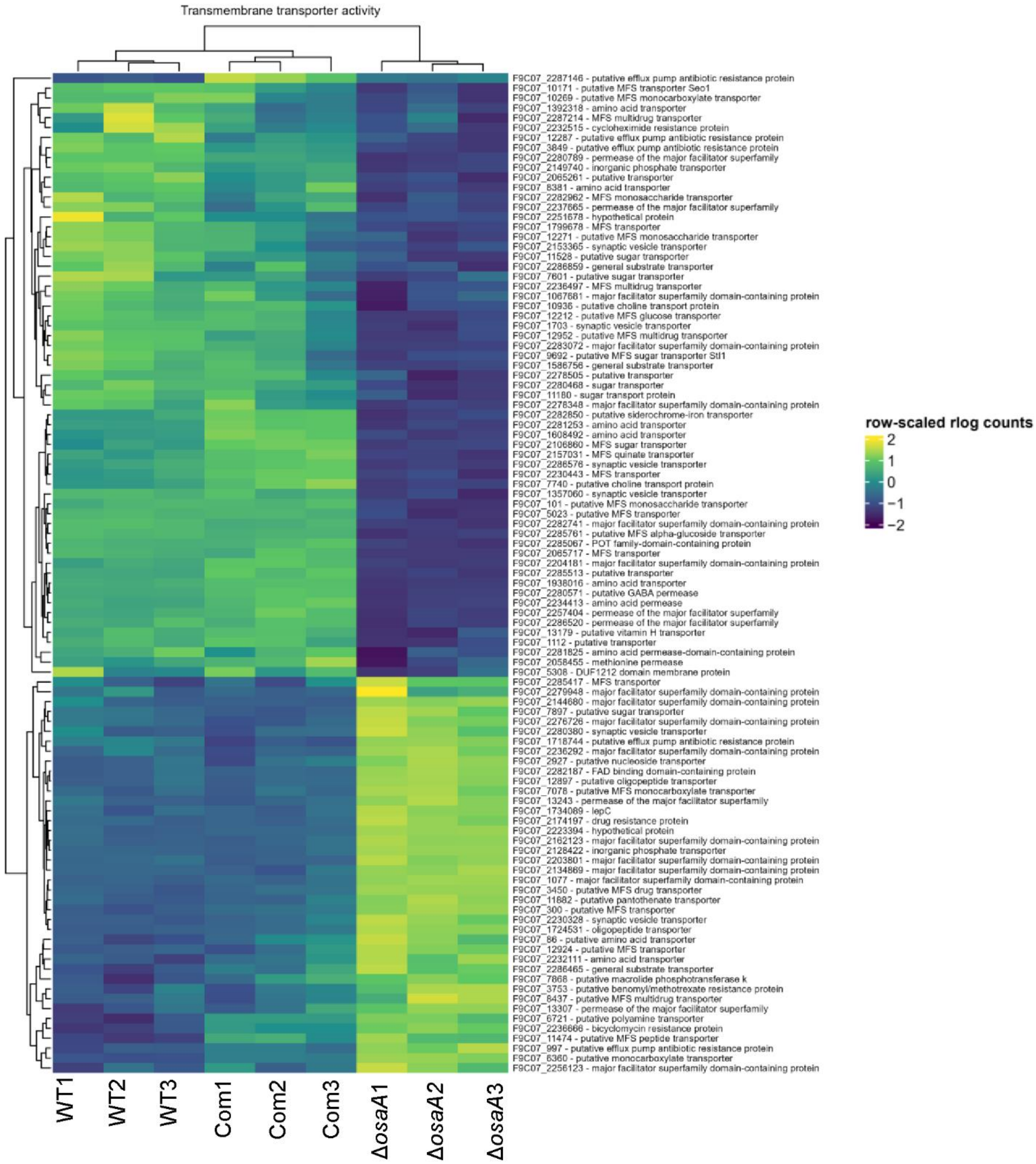

Figure S7. Differential expression of transmembrane transporter genes. Heatmap representation of transporter gene expression profiles in wild type (WT), *osaA* deletion and complementation strains. Each row corresponds to a transporter gene, annotated by its locus tag and predicted function. Yellow indicates higher expression while dark blue indicates lower expression, with color intensity reflecting the magnitude of fold change values.

Table S1: Primers used in this study

Primer No	Primer Name	Sequence
3011	Afl_OsaA_P1	CGGTGGTATGTCTCTCCAGTTCG
3012	Afl_OsaA_P2	TGTTTATGGTAGTTGTAGGTTCGAACCTAG
3013	Afl_OsaA_P3	TCATTGTTGATCCAGCCCCTCTAC
3014	Afl_OsaA_P4	CCCCCCAGTCAATAAGACCCC
3015	Afl_OsaA_P5	CTAGGTTCGAACCTACAACTACCATAAACAaccggtcgcctcaaacaatgctct
3016	Afl_OsaA_P6	GTAGAGGGGCTGGATCAACAATGAgtctgagaggaggcactgatgcgt
3017	Afl_OsaA_P7	CCG GTC ACT CTT CAC GGT CTG G
3018	Afl_OsaA_P8	GGG GAA GAG AGA GGG GAG ACC C
3046	osaA _Afl_F	ATGGTCAACGGCACCACCA
3047	osaA _Afl_R	CGCCTCCGGTGTAATAAGGGGTAG
2846	pyrG_AfumR	GAGCAGCGTAGATGCCTCGAC
2871	pyrG_Afum_F	ACCGGTCGCCTCAAACAATGCTCT
2872	pyrG_Afum_R	GTCTGAGAGGAGGCACTGATGCG
2869	trpC F	CCCATGTCAACAAGAATAAAACGC
2870	trpC R	CCGAGTGGAGATGTGGAGT
3071	Afl_osaA_qPCR_F	CGTTGTACGGATATCGTCCTCCGC
3072	Afl_osaA_qPCR_R	GGATTGATCCGGCCATTGAATACGCC
3063	Afl_osaA_P1 com fuse	CGGTGGTATGTCTCTCCAGTTCG
3064	Afl_osaA_pyrG R com fuse	GGGACCTTCCATATTAAGCTATCAGTCC
3065	Afl_osaA_com2F	GAGATGATGATCCGATGACTTGGTCG
3066	Afl_osaA-com2R_trpC linker	GCGTTTTATTCTTGTTGACATGGGTTATACCTGCGGTTGAGCTGGAGC
3067	trpC linker_PtrA_F	ACTCCACATCTCCACTCGGATGGGGTGACGATGAGCCGC
3068	PtrA R_ PyrG linker	AGAGCATTGTTTGAGGCGACCGGTGGGCAATTGATTACGGGATCCCAT

Table S2: All differentially expressed gene (DEG) and their log2 fold change (excel file)

Table S3: Enriched annotation terms in differentially expressed genes in the *osaA* deletion vs wild type comparison

Direction	Term	Annotation_category	pvalue_adjusted	n_DEGS	n_category
down	Apoplastic	ApoplastP	0	96	470
down	Apoplastic effector	EffectorP	0.0000001	38	146
down	membrane [GO:0016020]	Gene Ontology (cellular component)	0.0000288	224	1953
down	extracellular region [GO:0005576]	Gene Ontology (cellular component)	0.0009543	27	127
down	Cytoplasmic effector	EffectorP	0.0207458	19	92
down	cutinase activity [GO:0050525]	Gene Ontology (molecular function)	0.0247675	4	5
down	Pentose phosphate pathway	kegg_pathway	0.0294456	9	28
up	smurf_cluster_54-Aflatoxin	smurf_cluster	0	26	30
up	Aflatoxin biosynthesis	kegg_pathway	0	15	19
up	smurf_cluster_21-imizoquins	smurf_cluster	0.0000001	8	9
up	smurf_cluster_21-Aspirochlorine	smurf_cluster	0.0000488	8	15
up	monooxygenase activity [GO:0004497]	Gene Ontology (molecular function)	0.0003183	23	152
up	phosphopantetheine binding [GO:0031177]	Gene Ontology (molecular function)	0.0007182	11	41
up	secondary metabolite biosynthetic process [GO:0044550]	Gene Ontology (biological process)	0.0008737	23	163
up	O-methyltransferase activity [GO:0008171]	Gene Ontology (molecular function)	0.0009379	9	28
up	Cytoplasmic/apoplastic effector	EffectorP	0.0009655	8	22
up	Apoplastic	ApoplastP	0.0014787	45	470
up	smurf_cluster_22a	smurf_cluster	0.0043961	4	5
up	iron ion binding [GO:0005506]	Gene Ontology (molecular function)	0.0058798	20	151
up	oxidoreductase activity, acting on paired donors, with incorporation or reduction of molecular oxygen [GO:0016705]	Gene Ontology (molecular function)	0.0058798	17	116
up	PATHWAY: Mycotoxin biosynthesis. {ECO:0000256|ARBA:ARBA00004685}.	Pathway	0.0075154	5	10
up	organic cyclic compound biosynthetic process [GO:1901362]	Gene Ontology (biological process)	0.0081712	17	120
up	mycotoxin biosynthetic process [GO:0043386]	Gene Ontology (biological process)	0.0093544	7	23
up	heme binding [GO:0020037]	Gene Ontology (molecular function)	0.0212149	19	156
up	oxidoreductase activity [GO:0016491]	Gene Ontology (molecular function)	0.0263786	43	510
up	Cytochrome P450	kegg_pathway	0.0272698	6	20
up	smurf_cluster_55-Cyclopiazonic acid	smurf_cluster	0.0407695	3	4

Table S4: Differentially expressed gene (DEG) of aflatoxin biosynthetic gene cluster annotations and their log2 fold change (excel file)
